# Tailored Solid Polymer Electrolytes by Montmorillonite with High Ionic Conductivity for Lithium-Ion Batteries

**DOI:** 10.1186/s11671-019-3210-9

**Published:** 2019-12-05

**Authors:** Yingjian Zhao, Yong Wang

**Affiliations:** 1 State Key Laboratory of Electronic Thin Films and Integrated Devices, University of Electronic Science and Technology of China, Chengdu, 610054 China; 2 Chengdu No.7 High School, Chengdu, 610041 China

**Keywords:** Solid polymer electrolyte, Lithium-ion battery, Electrochemical window, Montmorillonite, Lewis acid-base theory

## Abstract

Polyethylene oxide (PEO)-based solid polymer electrolytes (SPEs) have important significance for the development of next-generation rechargeable lithium-ion batteries. However, strong coordination between lithium ions and PEO chains results the ion conductivity usually lower than the expectation. In this study, sub-micron montmorillonite is incorporated into the PEO frames as Lewis base center which enables the lithium ions to escape the restraint of PEO chains. After involving montmorillonite (MMT) into the SPEs, the ionic conductivity of SPEs is 4.7 mS cm^− 1^ at 70 °C which shows a comparable value with that of liquid electrolyte. As coupling with LiFePO_4_ material, the battery delivers a high discharge capacity of 150.3 mAh g^− 1^ and an excellent rate performance with a capacity of 111.8 mAh g^− 1^ at 0.16 C and maintains 58.2 mAh g^− 1^ at 0.8 C. This study suggests that the customized incorporation of Lewis base materials could offer a promising solution for achieving high-performance PEO-based solid-state electrolyte.

## Introduction

The requirements of energy storage devices for portable electronic [1], communication equipment [2], and hybrid electric vehicles are emerging [2–4]. Typically, the storage devices are proposed using lithium-ion batteries (LIBs), which have high specific energy, light weight, and easy to carry and quick to set up, as power sources to meet those fields [5–11]. However, for the commercial lithium-ion batteries, the liquid electrolyte systems suffer huge threats due to the flammability and the effects of a poison [5, 12, 13]. For instance, the boiling point of ethyl acetate, dimethyl carbonate, diethyl carbonate, and ethylene carbonate is only 77 °C, 90 °C, 127 °C, and 243 °C, respectively [5]. More importantly, the component material of commercial separators is polyethylene (PE) or polypropylene (PP), which will deform as the temperature up to 60 °C [14]. Therefore, once the operating temperature (> 60 °C) exceeds the critical temperature, the structure of separators will shrivel, resulting in inner short due to the lost function for physical dividing the cathode and anode [14, 15]. As compared, the solid electrolytes are worth expectation, they have the most competitive strategies to battle abovementioned issues because of thermal stability, chemical durability, and electrochemistry compatibility [16–19].

The inorganic solid electrolytes, such as sulfides (e.g., Li_10_GeP_2_S_12_ [20], Li_9.54_Si_1.74_P_1.44_S_11.7_Cl_0.3_ (25 mS cm^− 1^) [21], Li_11_Si_2_PS_12_ [22]), oxides (e.g., Li_7+2x−y_(La_3−x_Rb_x_)(Zr_2−y_Ta_y_)O_12_ (0 ≤ x ≤ 0.375, 0 ≤ y ≤ 1) [23], and Li_7_La_3_Zr_2_O_12_ [18]), show an exceptionally high conductivity. Some researchers reported that the lithium ion conductivity can reach up to 25 mS cm^− 1^, which is far higher than the conductivity of liquid electrolyte (~ 10^− 3^ S cm^− 1^) [21]. However, for inorganic solid electrolytes, they show the poor mechanical properties with low Young’s modulus and large number of grain boundaries within the inner of solid electrolyte [24], resulting in the failure for scale production [1].

Inorganic solid electrolyte combining with ion conductive polymer polyethylene oxide (PEO) has attracted widespread concern for solid polymer electrolytes (SPEs) to overcome abovementioned issues due to the unique features that PEO has excellent mechanical stability, reliable film forming capability, especially, the good compatibility with the lithium metal anode [17, 25, 26]. However, due to the Lewis base performance of PEO, lithium ions tend to imprison on the PEO chains, resulting in low lithium ion conductivity [17, 27–29].

In this work, we introduce a small quantity of sub-micro montmorillonite as a Lewis base center into the SPEs where the montmorillonite can establish the coordinate with lithium ions because the montmorillonite serves as a competitor to compete the lithium ions [30]. As a result, the proposed SPEs deliver high ionic conductivity (4.7 mS cm^− 1^) at 70 °C and the prepared all solid lithium-ion battery coupling LiFePO_4_ as cathode contributes discharge capacity of 150.3 mAh g^− 1^ with the LiFePO_4_ loading of 2 mg cm^− 2^, far exceeding the PEO-based solid electrolyte (119.1 mAh g^− 1^) at a current density of 0.08 C (1 C = 0.170 mAh g^− 1^).

## Experimental Methods

### Materials and Chemicals

For solid polymer electrolyte preparation, 500 mg PEO (Aladdin) and 250 mg lithium bis(trifluoromethanesulfonyl)imide (LITSFI, Aladdin) are dissolved into 10 mL acetonitrile (Aladdin), and then, 150 mg Li_6.4_La_3_Zr_1.4_Ta_0.6_O_12_ (LLZTO, Tai’an Faraday Energy Technology Co., Ltd) is added into the PEO solution with fast stirring at 70 °C to ensure uniform distribution. Finally, the slurry is casted on the surface of the Teflon film and dried at 80 °C under Ar atmosphere. For comparison, the MMT-based solid electrolyte is prepared using same method except the montmorillonite (Aladdin) is additional added with the mass loading of 100 mg.

### Characterization

Thermogravimetric (TG, Netzsch STA 449F3) analysis is performed for thermal stability with a heating rate of 10 °C min^− 1^ at Ar atmosphere. The crystal structure is confirmed via X-ray diffraction (XRD) patterns at room temperature using a UltimaIV diffractometer with CuKα1 radiation (*λ* = 1.4506 Å) and a position-sensitive detector. The surface morphologies and corresponding energy-dispersive X-ray (EDX) of the SPEs are observed by scanning electron microscope (SEM, FEI NANOSEI 450).

### Electrochemical Measurements

All electrochemical tests are conducted with standard coin cell (CR 2025). AC impedance spectroscopy is carried out by electrochemical workstation (CHI660E, Chenhua Instruments Co., China) at a frequency region of 0.1 Hz–100 MHz. Linear sweep voltammetry (LSV, 2.5 to 6.0 V with the scan rate of 10 mV^− 1^) and cyclic voltammetry (CV, − 0.5 to 6.0 V with the scan rate of 10 mV^− 1^) are conducted on the electrochemical workstation (CHI660E, Chenhua Instruments Co., China) with a stainless steel as working electrode and Li metal as a reference and counter electrode. The cycles are carried out by CT2001A cell test instrument (Wuhan LAND Electronic Co, Ltd). Coin cells sandwiching the SPEs between two stainless steel electrodes are assembled for lithium ion conductivity, which is calculated according to Eq. (1).

1 $$ \upsigma =\frac{d}{\mathrm{RA}} $$

where σ is conductivity, *d* is the thickness of SPEs, *R* is the resistance according to Nyquist plots, and *A* is the cross-section area. All solid-state lithium-ion batteries are assembled with LiFePO_4_ cathode coupling with lithium metal anode. Typically, LiFePO_4_, acetylene black, and polyvinylidene fluoride (7:2:1) are mixed with N-methyl-2-pyrrolidone (NMP). The mixture is coated on the aluminum foil and dried at 60 °C by vacuum overnight. The LiFePO_4_ loading in the cathode is 2 mg cm^− 2^.

## Results and Discussion

**Fig. 1:**
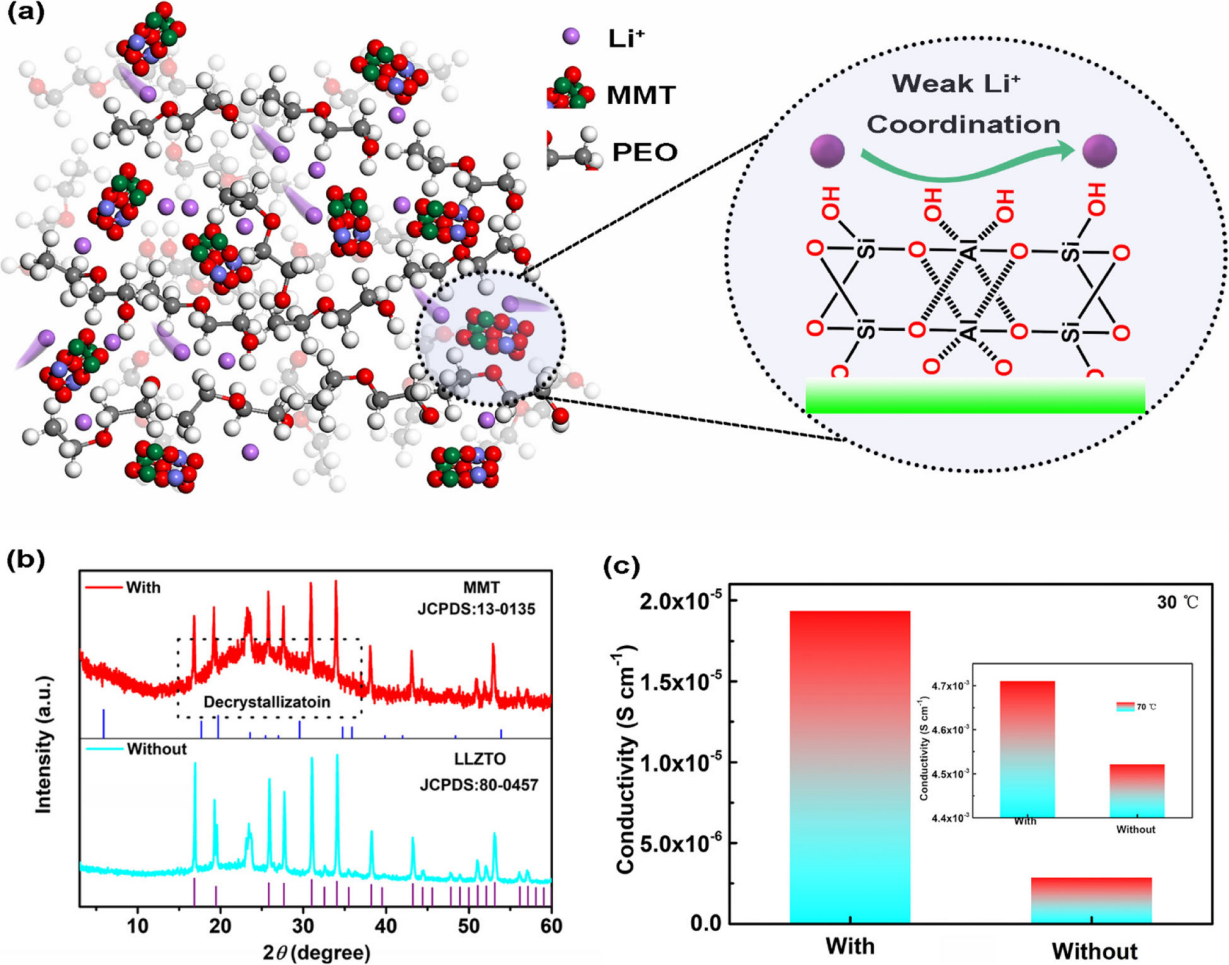
Characterization of SPEs with montmorillonite doping: **a** The design concept that lithium ion can fast diffusion along the surface of montmorillonite. **b**, **c** The XRD and FTIR results of SPEs with or without montmorillonite particles, respectively

To illustrate the relation of lithium ion diffusivities in a Lewis base environment, the design concept is shown in Fig. 1a, in which a small amount of montmorillonite as Lewis base center is added into the PEO frames. Based on Lewis acid-base theory, the montmorillonite can perform as a contender with PEO chain to allow the lithium ion (Lewis acid) self-concentrated on the surface of montmorillonite due to the high absorbing energy [14]; thus, the lithium ions can escape the restraint of PEO chains. Furtherly, the low lithium-ion diffusion energy barrier (0.15 eV) on the surface of montmorillonite can enable the lithium-ion migration freely because the strategies for facilitating ion transport such as decreasing the lithium-ion diffusion energy barrier by introducing fast ion conductor are high necessary [30]. As presented in Fig. 1b, according to the results derived from its XRD curve, a hill-like peak can be observed, implying the crystallinity of PEO has been decreased to some degree, which confirmed the ability of montmorillonite to weaken lithium ion coordination with PEO chains. Carried the ionic conductivity farther is tested via AC impedance spectroscopy where coin cells are sandwiched the SPEs between two stainless steel electrodes. As shown in Fig. 1c, the results clearly demonstrate the advantage after montmorillonite incorporation that the ionic conductivity of SPEs could be greatly improved. Specially, the ionic conductivity (4.7 mS cm^− 1^) of SPEs with montmorillonite incorporation at 70 °C is comparable with that of liquid electrolyte and would lead to the rapid transport of lithium ions.

**Fig. 2:**
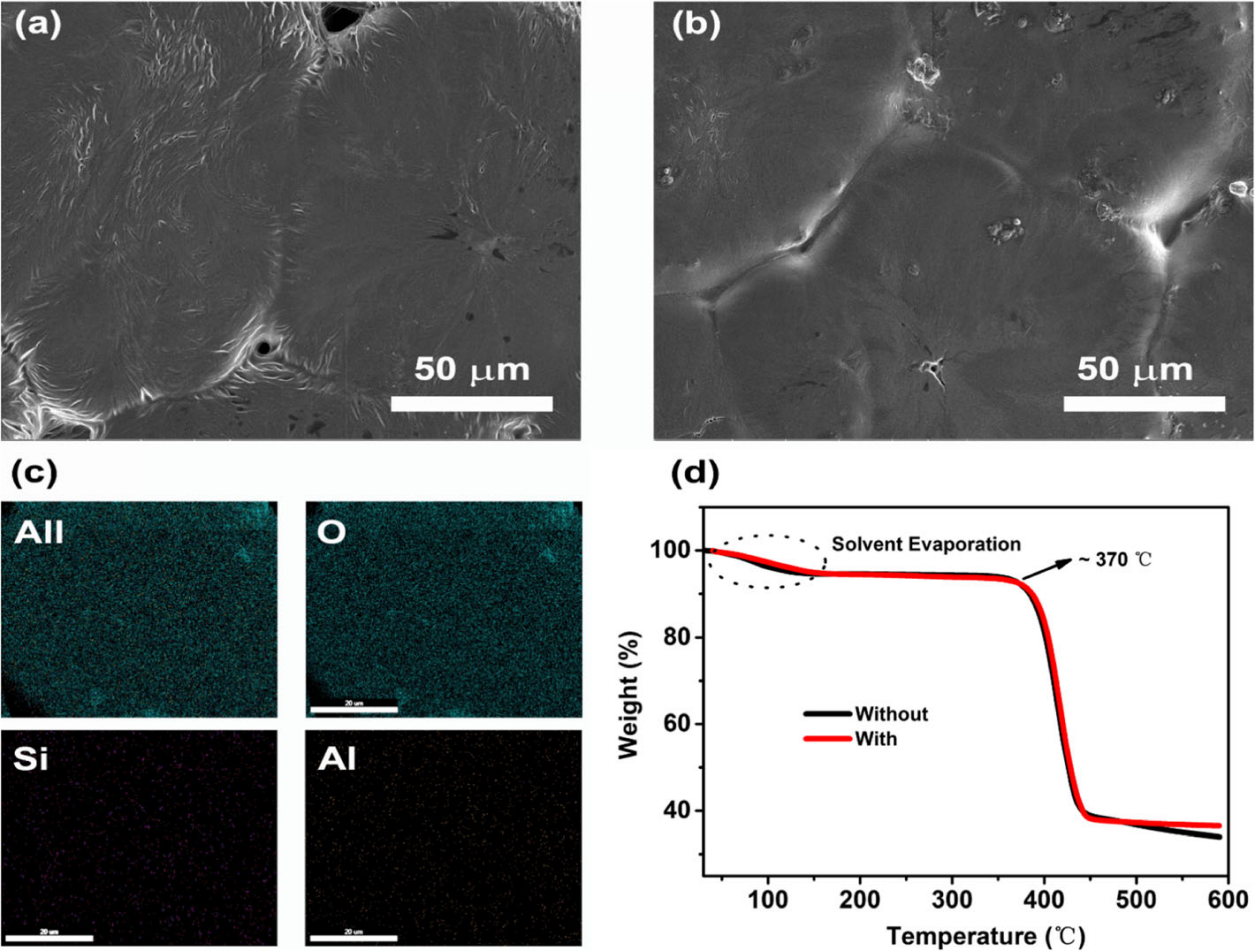
SEM images of SPEs without (**a**) and with (**b**) montmorillonite doping. **c** The element mapping of SPEs with modification of montmorillonite. **d** TGA curve of SPEs from 30 to 600 °C at a rate of 10 °C min^− 1^

Figure 2 presents the typical surface morphologies of the as-prepared SPEs. As shown in Fig. 2a, the SPEs without montmorillonite display uniform surfaces. The integrity of SPEs, however, was segmented into various irregular areas which may be caused by the solvent evaporation. Thereby, this structure increases the internal crystal interface of SPEs and slows down the transport of lithium ions. As contrast, this situation has been greatly optimized after montmorillonite involved in. The results show that the gaps between segmented SPEs have been filled due to the de-crystallization, presented in Fig. 1b. Furthermore, the feature element mapping of Si and Al confirmed the homogeneous distribution of montmorillonite particles embedded in the PEO matrix (Fig. 2c). Figure 2d shows the high-temperature performance of SPEs via thermogravimetric analysis. At low temperatures (< 150 °C), we observed a slight drop in weight, possibly from the evaporation of the residual solvent. Clearly, with or without montmorillonite, both of the SPEs present excellent thermal stability up to 370 °C.

**Fig. 3:**
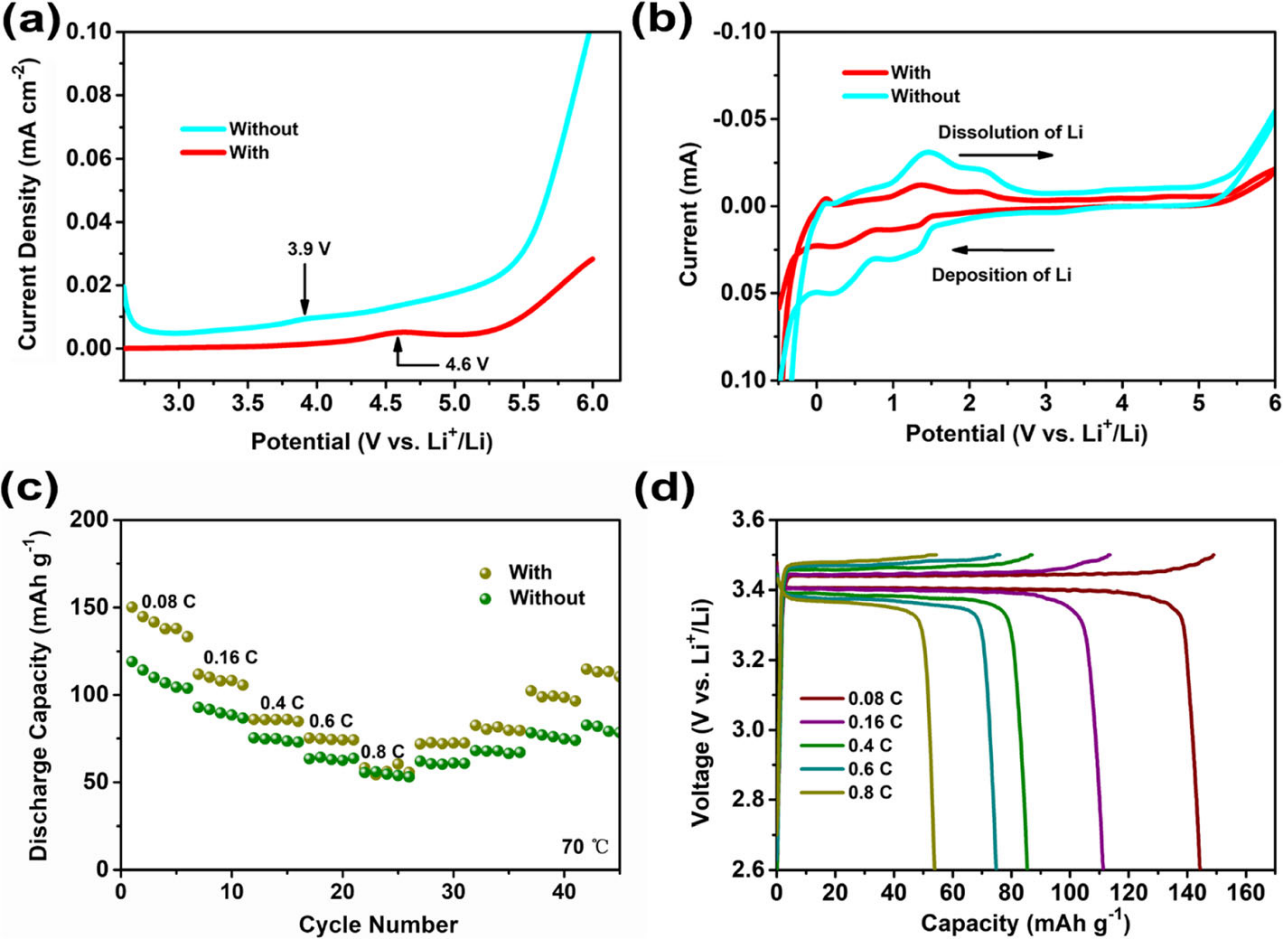
The electrochemical performance of SPEs: LSV profiles (**a**), cycling performance (**b**), rate performance (**c**), and voltage profiles of SPEs after montmorillonite (**d**)

Figure 3 presents the investigation of the electrochemical performances of SPEs. As shown in Fig. 3a, linear sweep voltammetry is employed to study the electrochemical window of SPEs before and after montmorillonite incorporation. Without montmorillonite, the oxidation process commences at 3.9 V. While the sweep can be extended to 4.6 V without an obvious current in the case after montmorillonite incorporation. The enhanced electrochemical stability can be attributed to the removed impurities such as water from the interface by the montmorillonite [31]. Correspondingly, the enhanced electrochemical stability is further confirmed via cyclic voltammetry (CV) scans that show the SPEs with montmorillonite deliver negligible redox current from 2.5 to 5 V (Fig. 3b). However, a contrasted phenomenon has been observed that the SPEs without montmorillonite increases the oxidation current, consisting with LSV results. Furtherly, the galvanostatic charging and discharging cycles of LiFePO_4_ batteries are tested at 70 °C to confirm the actual applications of SPEs. As shown in Fig. 3c, the specific discharge capacity is 150.3 mAh g^− 1^ with a high Coulombic efficiencies nearly 100% at 0.08 C, which is 88% of the theoretical value (170 mAh g^− 1^). Corresponding, the typical potential plateaus of LFP at 3.39 V and 3.44 V corresponding to discharge and charge can be clearly identified. As the current densities are increased to 0.16, 0.4, 0.6, and 0.8 C, the specific discharge capacities diminish to 111.8, 85.9, 75.2, and 58.2 mAh g^− 1^, respectively. Without montmorillonite, lower discharge capacity could be found as only 119.1 mAh g^− 1^ at 0.08 C, which is 70% of the theoretical value. As the current density increases, the specific discharge capacities fast decrease to 92.8, 75.4, 63.4, and 55.5 mAh g^− 1^ corresponding to 0.16, 0.4, 0.6, and 0.8 C, respectively. Therefore, all of the results clearly demonstrate again the benefits of montmorillonite to tailor all solid-state electrolyte with high ionic conductivity for actual application of lithium-ion batteries.

## Conclusions

In summary, a small amount of montmorillonite as Lewis base center is added into the PEO frames to enable SPEs achieving high ionic conductive. The uniformly distribution of montmorillonite allows electrochemical window of SPEs improved from 3.9 to 4.6 V. This proposed strategy exhibits an excellent electrochemical performance that the prepared LiFePO_4_ battery delivers a high discharge capacity of 150.3 mAh g^− 1^ with the loading of 2 mg cm^− 2^ at 70 °C, far exceeding the control sample (119.1 mAh g^− 1^) at a same current density of 0.08 C. All the results indicate the proposed strategy based on Lewis acid-base theory could be a promising method to achieve high-capacity and high-rate lithium-ion batteries.

## Data Availability

All data are fully available without restriction.

## Abbreviations

CV: Cyclic voltammetry
EDX: Energy-dispersive X-ray
LIBs: Lithium-ion batteries
LITFSI: Bis(trifluoromethanesulfonyl)imide
LLZTO: Li_6.4_La_3_Zr_1.4_Ta_0.6_O_12_
LSV: Linear sweep voltammetry
MMT: Montmorillonite
NMP: N-methyl-2-pyrrolidone
PE: Polyethylene
PEO: Polyethylene oxide
PP: Polypropylene
SEM: Scanning electron microscope
SPEs: Solid polymer electrolytes
TG: Thermo-gravimetric
XRD: X-ray diffraction
